# Case Report: Multifocal cerebral infarctions caused by cyanoacrylate glue embolization after gastric variceal injection

**DOI:** 10.3389/fmed.2026.1825258

**Published:** 2026-05-18

**Authors:** Ahmed Hafez Mousa, Samar Iltaf Mairajuddin, Anas Alnajjar, Ahmed Aramram, Abdullah AlKurdi, Youssef ElSabban, Doaa Zain Fadaaq, Ayoub Ahmed Abedzadeh, Shaikh Sayeed Iqbal, Abubaker Abdul Rahman Shaffi Al Madani, Babacar Cisse

**Affiliations:** 1 Neurosciences, Dubai Health, Dubai, United Arab Emirates; 2 Department of Neurosurgery, Graduate Medical Education, Mohammed Bin Rashid University of Medicine and Health Sciences, Dubai Health, Dubai, United Arab Emirates; 3 Department of Radiology, Graduate Medical Education, Mohammed Bin Rashid University of Medicine and Health Sciences, Dubai Health, Dubai, United Arab Emirates; 4 Department of Internal Medicine, Dubai Health, Dubai, United Arab Emirates; 5 College of Medicine, Mohammed Bin Rashid University of Medicine and Health Sciences, Dubai Health, Dubai, United Arab Emirates; 6 College of Medicine and Surgery, Batterjee Medical College, Jeddah, Saudi Arabia

**Keywords:** case report, cerebral embolism, cyanoacrylate, gastric varices, portal hypertension, portopulmonary venous anastomosis

## Abstract

**Background:**

Endoscopic injection of N-butyl-2-cyanoacrylate (Histoacryl) is a first-line treatment for bleeding gastric varices. Although generally safe, rare systemic complications including paradoxical cerebral embolization can occur, carrying devastating neurological consequences.

**Case presentation:**

We report a 62-year-old man with probable alcohol-related cirrhosis who presented with acute upper gastrointestinal bleeding secondary to gastroesophageal gastric varices and underwent urgent endoscopic cyanoacrylate injection with a total volume of 8 mL, achieving immediate hemostasis. Post-procedure, the patient failed to regain consciousness. Neuroimaging revealed extensive multifocal cerebral embolization with radiopaque glue material involving both anterior and posterior circulation territories, consistent with systemic cyanoacrylate embolization. Transesophageal echocardiography with agitated saline contrast confirmed the absence of a patent foramen ovale or intracardiac right-to-left shunt, supporting an extracardiac mechanism of embolization, most probably via portopulmonary venous anastomoses related to portal hypertension. Despite intensive neurocritical care, the patient developed progressive cerebral edema with herniation and died 48 h later.

**Conclusion:**

Cerebral embolization is a rare but catastrophic complication of endoscopic cyanoacrylate injection. This case highlights the role of unrecognized portosystemic collateral pathways in facilitating paradoxical embolization even in the absence of a cardiac shunt. Failure to awaken or acute neurological deterioration post-variceal therapy should prompt immediate neuroimaging. Greater emphasis on pre-procedural imaging, careful injection technique, and risk-reduction strategies is essential.

## Introduction

Gastric variceal bleeding is a severe and potentially life-threatening complication of portal hypertension, associated with higher morbidity, mortality, and rebleeding rates compared with esophageal varices. Endoscopic injection of N-butyl-2-cyanoacrylate (Histoacryl) has become a well-established first-line therapy for bleeding gastric varices, particularly gastroesophageal varices type 2 (GOV2) and isolated gastric varices, due to its high efficacy in achieving rapid hemostasis and reducing early rebleeding (1, 2). Despite its widespread use and overall favorable safety profile, cyanoacrylate injection is associated with rare but devastating systemic complications (3).

Among these, cerebral embolization represents one of the most catastrophic outcomes, often resulting in multifocal ischemic strokes, severe neurological impairment, or death. The proposed mechanisms include paradoxical embolization through intracardiac right-to-left shunts such as a patent foramen ovale, as well as extra-cardiac pathways related to portal hypertension, including spontaneous portosystemic and portopulmonary venous shunts. Because these vascular collaterals may be clinically silent and underrecognized, the risk of systemic embolization may not be fully appreciated at the time of endoscopic intervention (4, 5).

Reported cases of cerebral embolism following cyanoacrylate injection are exceedingly rare, and current evidence is largely limited to isolated case reports and small case series. Consequently, there is no standardized strategy for risk stratification, prevention, or management once embolization occurs, and outcomes are frequently poor. Early recognition remains challenging, particularly when neurological deterioration occurs immediately following sedation for endoscopy and may initially be misattributed to anesthetic effects (6, 7).

We present a fatal case of extensive multifocal cerebral infarction secondary to systemic embolization of cyanoacrylate following endoscopic treatment of bleeding gastric varices in a patient with presumed alcohol-related cirrhosis and no identifiable intracardiac shunt. This case highlights the critical role of occult portosystemic collateral pathways in facilitating paradoxical embolization, underscores the importance of early neuroimaging when delayed awakening or neurological decline occurs after variceal therapy, and reinforces the need for heightened awareness, preventive strategies, and careful procedural planning in patients undergoing cyanoacrylate injection for gastric varices.

## Case description

### Presentation and initial management

A 62-year-old man with a long-standing history of alcohol use disorder presented after 2 days of nausea and repeated episodes of hematemesis. He reported three episodes of moderate-volume vomiting containing blood clots, followed by pre-syncope and transient loss of consciousness at home. Shortly thereafter, he passed black, tarry stools consistent with melena. He had consumed alcohol heavily for approximately 20 years but had reduced his intake to occasional monthly drinking over the past 2 years. He had no known chronic illnesses, was not taking medications, and denied NSAID use. While visiting Dubai from Colombia, he had not experienced prior gastrointestinal bleeding, jaundice, pruritus, ascites, or encephalopathy.

He was initially taken to a private hospital, where he received unspecified intravenous medications before being transferred to a second facility. There, he was treated with intravenous octreotide, ceftriaxone, and 2 units of packed red blood cells. Upper endoscopy revealed mild esophageal varices without high-risk stigmata and large gastric varices (GOV2) in the fundus extending toward the cardia. Stigmata of recent bleeding, including red wale markings and a fresh adherent clot, were present. Active bleeding developed during the procedure. Epinephrine was injected into the surrounding erosive mucosa, and three hemostatic clips were placed on visible non-variceal vessels, but no definitive therapy for the gastric varices could be provided. The patient was therefore referred to our tertiary center for specialized management.

Upon arrival to our emergency department, the patient was hemodynamically stable. Blood pressure was 120/71 mmHg, pulse 70 beats per minute, respiratory rate 16 breaths per minute, temperature 36.5 °C, and oxygen saturation 99% on room air. Orthostatic measurements revealed no significant drop in blood pressure. He appeared alert, oriented, and in no distress. Examination was notable for a distended, obese abdomen with visible abdominal wall veins and a positive Castell’s sign. There was no jaundice, asterixis, edema, or focal neurological deficit.

Laboratory evaluation demonstrated hemoglobin 10.8 g/dL, platelets 107 × 10^9^/L, INR 1.6, bilirubin 1.24 mg/dL, albumin 3.1 g/dL, creatinine 0.77 mg/dL, and urea 57 mg/dL. Liver enzymes were within normal range. His clinical picture suggested probable alcohol-related cirrhosis with acute upper gastrointestinal bleeding. He was assessed as Child–Pugh A (score 6) with a MELD-3.0 score of 12.

He was admitted and initiated on terlipressin, intravenous ceftriaxone, and continuous pantoprazole infusion. A same-day urgent upper endoscopy was performed under monitored anesthesia care. Sedation included fentanyl 15 μg, midazolam 1 mg, and propofol 330 mg; the patient was not intubated.

### Endoscopic procedure and neurological deterioration

Endoscopic evaluation revealed a small esophageal varix without high-risk features, a 6-cm hiatal hernia with erosions, and combined GOV1 and GOV2 gastric varices. No active bleeding was seen. A total of 8 mL of N-butyl-2-cyanoacrylate (histoacryl) was injected into the gastric varices, achieving immediate hemostasis. Portal hypertensive gastropathy was noted, and three hemostatic clips from the previous procedure were seen. The duodenum appeared normal, and rapid urease testing was negative.

Following the procedure, the patient failed to regain consciousness. Initial assessment showed a Glasgow Coma Scale score of 5/15, with bilaterally reactive pupils and preserved hemodynamic and respiratory parameters. Naloxone (0.4 mg) and flumazenil (0.4 mg) were administered without improvement. Laboratory studies remained unchanged, and there was no evidence of hypoxia, hypotension, or metabolic disturbance. ICU and neurology services were urgently consulted. Transesophageal echocardiography with agitated saline (bubble) contrast was performed on the same day and showed no patent foramen ovale, no atrial septal defect, and no intracardiac right-to-left shunt.

### Neuroimaging findings

**Figure 1:**
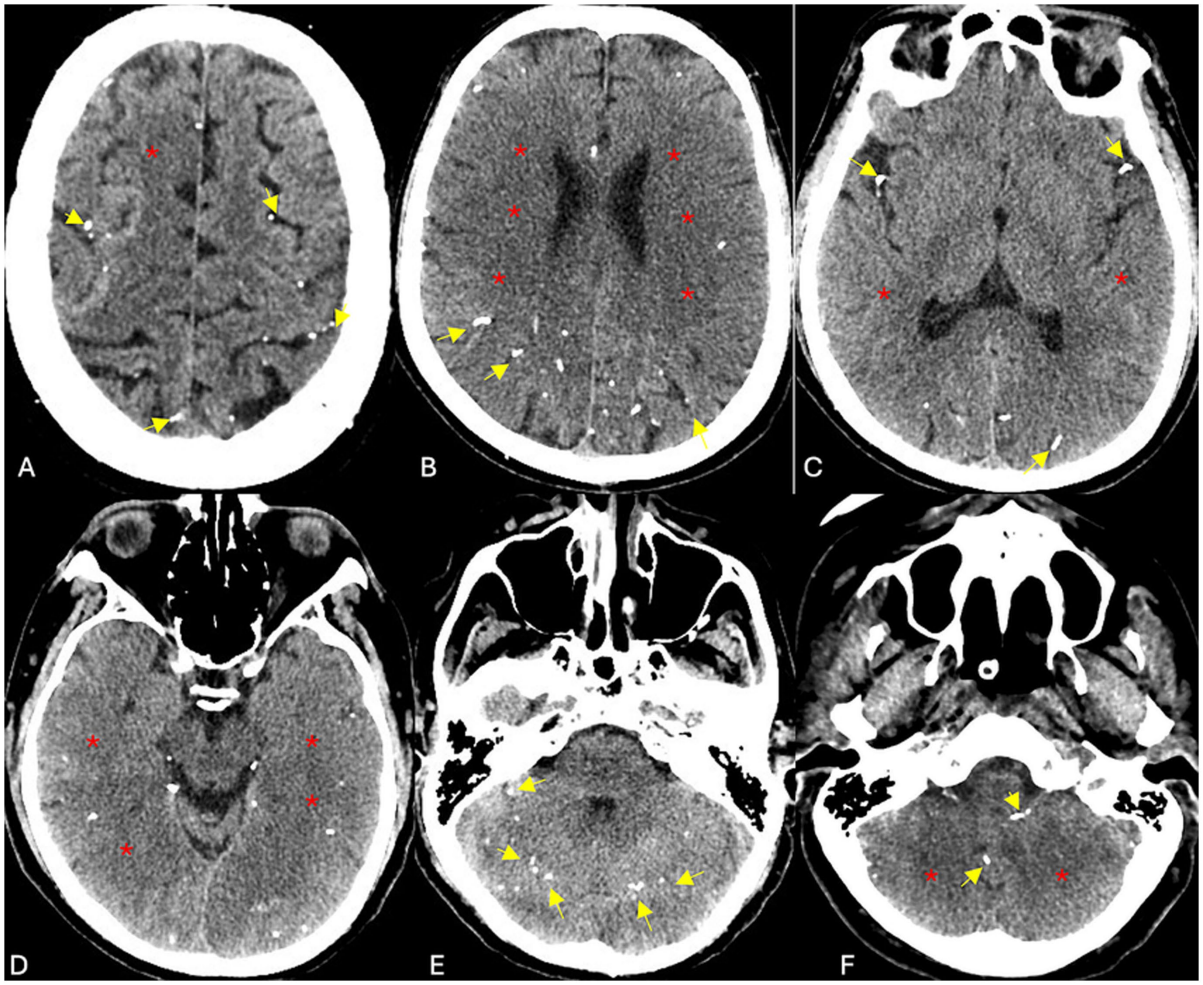
**(A–F)** Plain CT brain demonstrates multiple scattered hyperdense embolic foci (glue material) throughout the supratentorial compartment and posterior fossa, tracking along the major cerebral arteries and their peripheral and cortical branches (yellow arrows). Early acute ischemic changes are evident in both MCA territories, the right ACA territory, the watershed zones, and the bilateral PICA and PCA territories (red asterisks).

**Figure 2:**
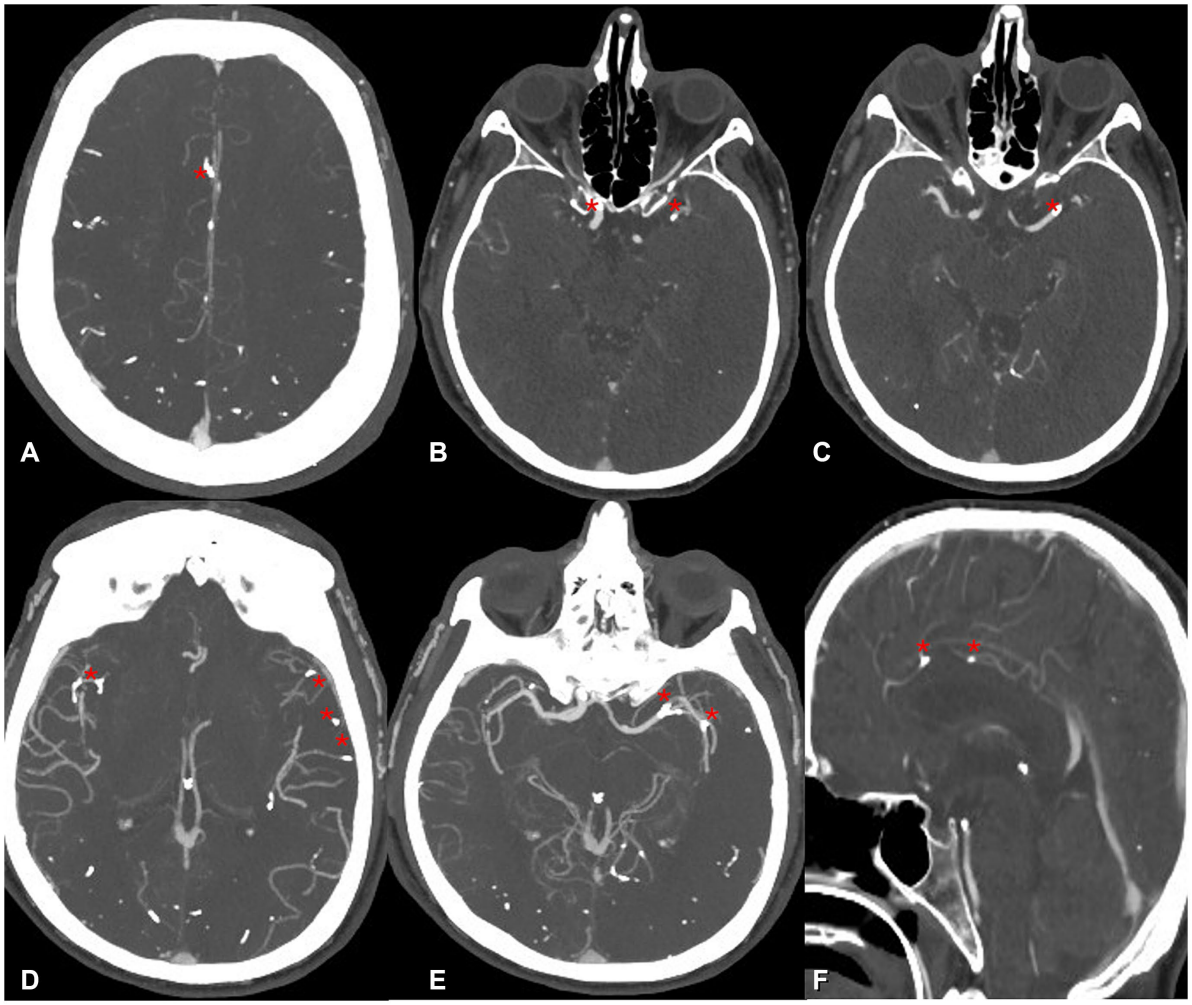
**(A–F)** Anterior circulation CT angiography shows multiple scattered hyperdense embolic foci (glue material) within the supraclinoid segment of the right internal carotid artery, left MCA M1 segment, and bilateral distal insular branches (M3–M4 segments). Additional embolic material is present within the right pericallosal and calloso-marginal branches of the right anterior cerebral artery (red asterisks).

**Figure 3:**
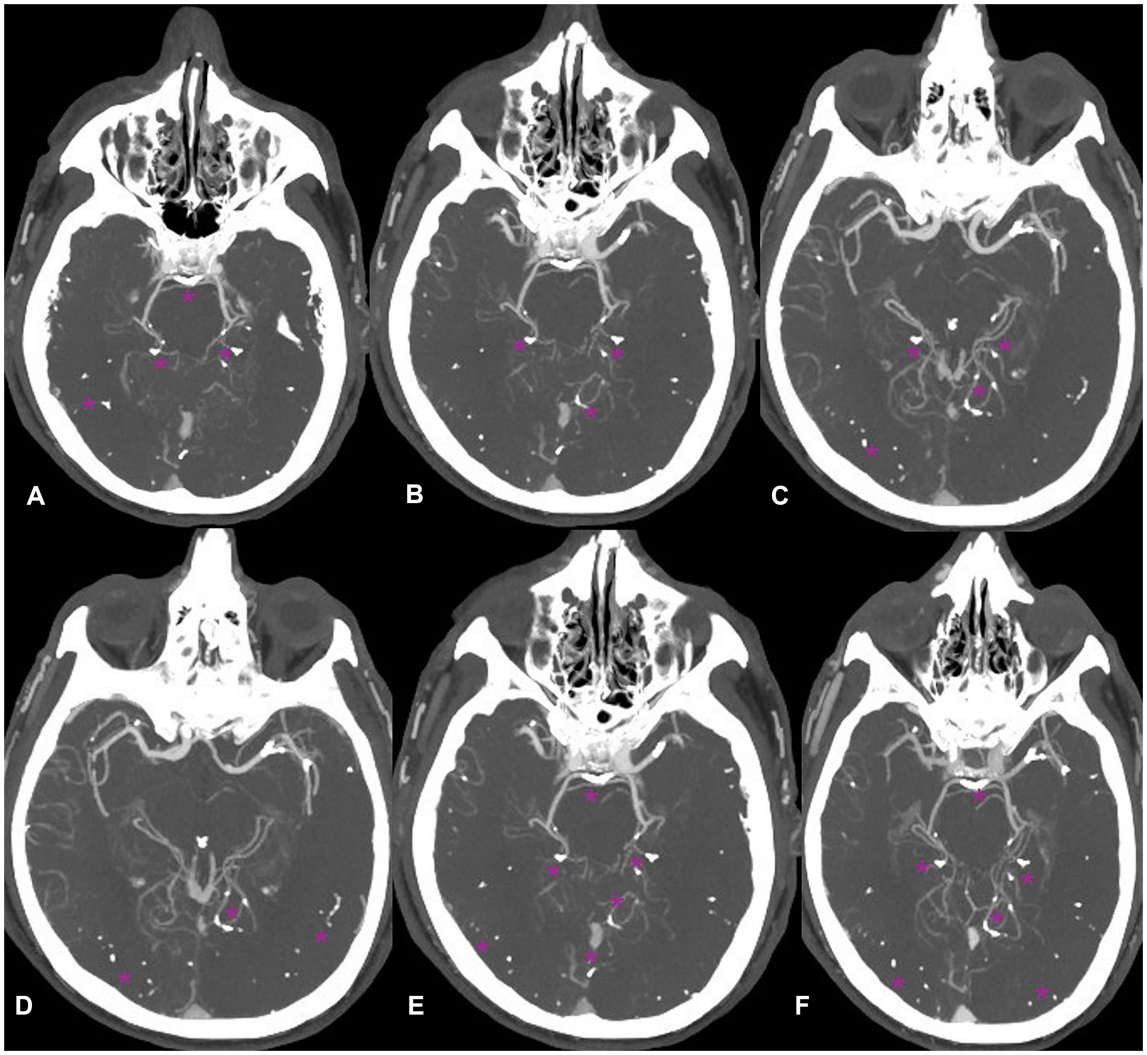
**(A–F)** Posterior circulation CT angiography demonstrates multiple scattered hyperdense embolic foci (glue material) involving the distal branches of both posterior inferior cerebellar arteries (PICA) and the origins of both posterior cerebral arteries (P1 segments), with extension into the P2–P4 cortical branches (purple asterisks).

Non-contrast CT of the brain followed by CT angiography revealed multiple radiopaque embolic foci distributed within the posterior fossa and supratentorial territories, involving branches of the anterior, middle, and posterior cerebral arteries (Figures 1–3). These findings were consistent with cerebral embolization of cyanoacrylate or Lipiodol used during variceal injection. Subtle early ischemic changes were noted in the bilateral frontoparietal and cerebellar regions, without large-vessel occlusion, intracranial hemorrhage, or midline shift. Given his recent gastrointestinal bleeding, non-thrombotic embolic material, and lack of an LVO target, thrombolysis and antithrombotic therapy were contraindicated. The patient was managed conservatively in the ICU with strict neurological monitoring and head elevation.

**Figure 4:**
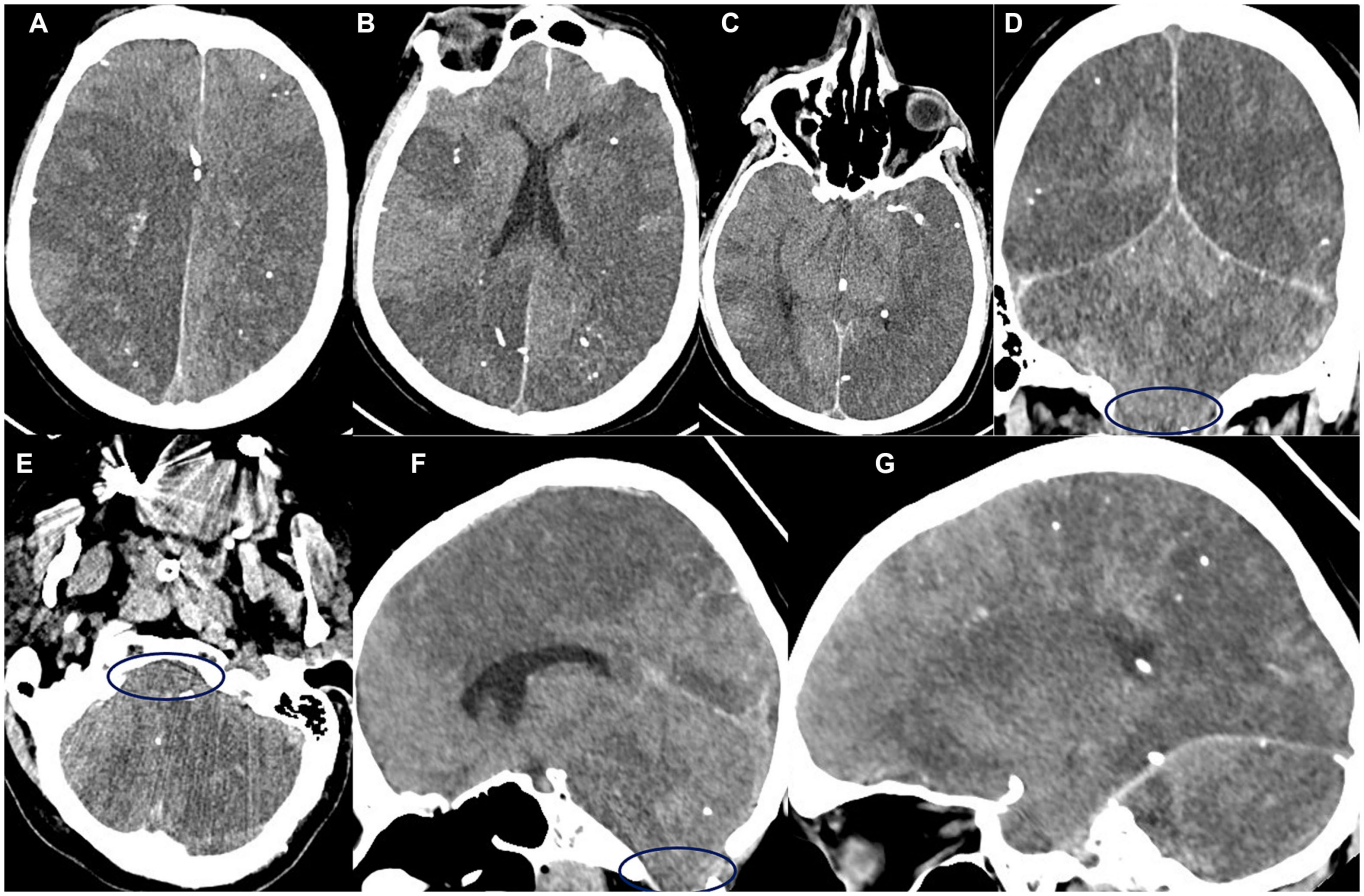
**(A–G)** Follow-up 24 h later non-contrast CT shows interval evolution of established infarcts involving the bilateral MCA territories, right ACA territory, and bilateral PCA and PICA territories. There is a significant worsening of cerebral oedema, resulting in downward cerebellar tonsillar herniation (blue circles). Multiple scattered hyperdense embolic foci (glue material) persist within the brain parenchyma.

A repeat CT scan performed 24 h later showed interval evolution of extensive infarctions involving bilateral middle cerebral artery (MCA), right anterior cerebral artery (ACA), bilateral posterior cerebral artery (PCA), and bilateral posterior inferior cerebellar artery (PICA) territories (Figure 4). There was marked progression of cerebral edema with early tonsillar herniation. Multiple hyperdense glue emboli persisted throughout the cerebral parenchyma. No intraparenchymal hemorrhage or hydrocephalus was present.

The patient remained in the intensive care unit under neurocritical care management for multifocal cerebral infarctions secondary to cyanoacrylate glue embolization following gastric variceal injection (Table 1).

**Table 1:** Clinical timeline from initial presentation to death.

Timepoint	Clinical event
Day 0 (Presentation)	62-year-old male with 2-day hematemesis and melena. Transferred after initial endoscopy at outside hospital showing GOV2 gastric varices with stigmata of recent bleeding. Hemodynamically stable on arrival. Child–Pugh A (score 6), MELD-3.0 score 12.
Day 0 (Endoscopy)	Urgent upper endoscopy under sedation (fentanyl 15 μg, midazolam 1 mg, propofol 330 mg); not intubated. GOV1 + GOV2 gastric varices identified. 8 mL N-butyl-2-cyanoacrylate injected. Immediate hemostasis achieved.
Day 0 (Post-procedure)	Failed to regain consciousness. GCS 5/15 with bilaterally reactive pupils. Naloxone 0.4 mg and flumazenil 0.4 mg given without improvement. No metabolic or hemodynamic derangement.
Day 0 (Post-procedure)	Transesophageal echocardiography with agitated saline (bubble) contrast: no PFO, no intracardiac right-to-left shunt, no structural cardiac abnormality.
Day 0 (~1 h post-procedure)	CT brain + CTA: multifocal radiopaque embolic foci across ACA, MCA, PCA, and PICA territories with early ischemic changes. Thrombolysis and antithrombotic therapy contraindicated given recent GI bleed.
Day 1 (24 h)	Repeat CT brain: extensive bilateral infarctions across MCA, ACA, PCA, and PICA territories. Progressive cerebral edema with early tonsillar herniation. Persistent glue emboli.
Day 2 (48 h)	Cardiac arrest with asystole. Resuscitation unsuccessful. Patient deceased.

## Discussion

Systemic cerebral embolization following endoscopic treatment of gastroesophageal varices (GOV) using N-butyl-2-cyanoacrylate (Histoacryl glue), though rare, represents a catastrophic and potentially life-threatening complication. Pathogenesis involves migration of Histoacryl glue into the systemic circulation, which can occur via right-to-left shunts across the heart as seen in patients with patent foramen ovale (PFO) or atrial septal defect (ASD), or, as suspected in our case, via spontaneous portosystemic shunting secondary to portal hypertension. This case highlights the catastrophic result of such a complication and advocates for increased awareness of collateral vascular pathways even in the absence of a cardiac shunt.

Incidence of cerebral embolism following Histoacryl glue is extremely rare. A large cohort study conducted by Liu-fang et al. retrospectively reported 635 patients who were treated with injecting Histoacryl glue. Only 1 case had cerebral embolism, suggesting an incidence of 0.1% (8). Another case series by Yujen et al., published in 2018, identified only 5 cases of cerebral embolization among the total number of studies in their review (9). The most commonly identified mechanism has been a right-to-left shunt via a PFO, leading to paradoxical embolism (1, 7, 10). However, several recent case reports identified other collateral pathways, including Porto-pulmonary venous anastomosis (PPVA), pulmonary arteriovenous shunt, spontaneous porto-renal shunt, and spontaneous porto-azygous shunt (1, 11–13, 17). In our patient, the mechanism was most consistent with portopulmonary venous anastomosis (PPVA). Transesophageal echocardiography with agitated saline contrast was performed on the day of the procedure and confirmed the absence of a patent foramen ovale, atrial septal defect, or any intracardiac right-to-left shunt. This rules out the most commonly reported embolic pathway for cyanoacrylate cerebral embolization. The rapid and widespread distribution of glue across both anterior and posterior circulation territories, combined with the negative TOE, points to a direct portopulmonary venous route. PPVA has been reported in 10–18% of portal hypertension patients in imaging-based studies (11, 14). In this pathway, glue travels from the portal system directly into pulmonary veins, bypasses the pulmonary capillary bed, and enters the left heart, from which it can reach the cerebral circulation. Typically, Histoacryl glue injected into gastric varices may travel to the lungs via portosystemic shunts, resulting in pulmonary glue embolism. The incidence of PPVA in portal hypertension patients is not universally established. One cohort study by A et al. in which PPVA was identified in 4 patients out of 17, showed a prevalence of 18%, approximately (11). Another study by Yasuhiro et al. found 9 patients out of 92, corresponding to almost 9.7% (14). However, other CT-based case series and reviews reported a rare risk of PPVA (3, 11).

In our patient, a CT brain with CT cerebral angiography was done within one hour following the procedure, and it showed already Histoacryl glue as radio-opaque foci across multiple brain territories as well as early ischemic changes in the MCA territory. The timing of the procedure and early ischemic changes noticed suggest direct communication between the portal and systemic pulmonary veins, bypassing the pulmonary capillary bed, and causing paradoxical systemic embolization, specifically cerebral embolization (18). Due to the patient’s condition and instability, he was kept in the intensive care unit with the neurology team and neurosurgery team on board. The patient was deemed unfit for reperfusion therapy or any neurosurgical intervention, and only supportive treatment with close neuro-observation was provided. Unfortunately, the patient died 48 h following repeated CT brain as he sustained cardiac arrest with asystole and could not be revived. There is no robust evidence to guide treatment in cerebral embolism secondary to Histoacryl glue. Since most patients present primarily with gastric bleed, thrombolysis is contraindicated. Maeda et al., described a case in which glue embolism was successfully retrieved via mechanical thrombectomy (20). However, our case showed multiple occluded cerebral arteries with distal segments, making mechanical thrombectomy futile. This necessitates more focus on prevention rather than treatment to prevent poor outcomes.

Both the American Academy of Gastroenterology and the American Association for the Study of Liver Disease recommend contrast-enhanced cross-sectional imaging before an endoscopic procedure to delineate portosystemic collateral anatomy and possible anomalies (7, 8). Yujen et al. (13) reported in a retrospective case series the need for pre-procedural angiographic studies to identify spontaneous portosystemic shunts. Guangchuan et al. (15) demonstrated a significant reduction in Histoacryl glue embolism incidence when a clip was applied before injecting Histoacryl glue. On the other hand, Seewald et al. focused on standardizing injection technique and regimen. His paper recommended a small total volume of injection, mixing ratio of 0.5 to 0.8 mL with Lipiodol, and a limited amount of injection to 1 mL at a time (10). In our case, 8 mL of cyanoacrylate was injected to achieve hemostasis in large combined GOV1 and GOV2 varices. This is well above the thresholds recommended by Seewald et al. (10). The high total volume likely contributed to the extensive cerebral embolization seen here. Clinical urgency sometimes demands larger volumes when bleeding cannot be controlled otherwise, but this case shows that the risk of systemic embolization increases with the amount injected. Adhering to volume-limitation protocols is important even when treating large or multiple varices. In cases where spontaneous portosystemic shunts were identified preoperatively, the studied measures have shown promising results in decreasing embolization risk, including Endoscopic ultrasound-guided injection of Histoacryl glue, balloon-occluded retrograde transvenous occlusion (BRTOcc), and fluoroscopic guidance (5, 16, 19). Following cerebral embolization with Histoacryl glue, treatment is mainly supportive, although mechanical thrombectomy has been successful in selected cases (9).

This case also raises the question of procedural counseling. Cyanoacrylate injection is often performed urgently, with limited time for detailed risk discussion. When clinical circumstances allow, however, patients and families should be informed of the rare but serious risk of systemic embolization, including cerebral embolism, as part of the consent process.

### Limitations

This case has several limitations. First, pre-procedural contrast-enhanced cross-sectional imaging was not obtained to identify portosystemic collaterals, including PPVA. The patient presented with acute bleeding and went directly to urgent endoscopy. Without direct anatomical demonstration of the PPVA pathway, the mechanism is inferred from the negative TOE and the pattern of embolization rather than proven by imaging. This gap supports the recommendation we make in this report: pre-procedural vascular imaging should be routine and might have changed the approach in this case. Second, this is a single case, and the observations cannot be generalized. Third, the patient was visiting Dubai from Colombia, and complete prior medical records were not available, limiting assessment of prior hepatic or cardiac history.

## Conclusion

Cerebral embolization after endoscopic cyanoacrylate injection for gastric varices is rare and almost always fatal. In this case, transesophageal echocardiography with bubble contrast excluded an intracardiac shunt, pointing to portopulmonary venous anastomoses as the embolic pathway. The 8 mL of cyanoacrylate injected exceeded recommended safety thresholds and may have worsened the extent of embolization. Pre-procedural vascular imaging to identify collateral pathways, careful adherence to volume limits during injection, and immediate neuroimaging when a patient fails to wake after endoscopy are the key preventive measures.

## Data Availability

The original contributions presented in the study are included in the article/supplementary material, further inquiries can be directed to the corresponding author.
